# Upregulation of the Sarco-Endoplasmic Reticulum Calcium ATPase 1 Truncated Isoform Plays a Pathogenic Role in Alzheimer’s Disease

**DOI:** 10.3390/cells8121539

**Published:** 2019-11-28

**Authors:** Renaud Bussiere, Bénédicte Oulès, Arnaud Mary, Loan Vaillant-Beuchot, Cécile Martin, Wejdane El Manaa, Déborah Vallée, Eric Duplan, Patrizia Paterlini-Bréchot, Cristine Alves Da Costa, Frédéric Checler, Mounia Chami

**Affiliations:** 1 Université Côte d’Azur, INSERM, CNRS, IPMC, France, Laboratory of excellence DistALZ, 660 route des Lucioles, 06560 Sophia-Antipolis, Valbonne, France; r.bussiere@imperial.ac.uk (R.B.); mary@ipmc.cnrs.fr (A.M.); vaillant@ipmc.cnrs.fr (L.V.-B.); ccilmart1@gmail.com (C.M.); elmanaa@ipmc.cnrs.fr (W.E.M.); deborah-vallee@hotmail.fr (D.V.); duplan@ipmc.cnrs.fr (E.D.); checler@ipmc.cnrs.fr (F.C.); 2 Present address: UK Dementia Research Institute, Imperial College London, Department of Medicine, Burlington Danes Building, Hammersmith Hospital Campus, Du Cane Road, London W12 0NN, UK; 3 Institut Cochin, Team Cutaneous Biology, INSERM U1016, CNRS UMR8104, Université Paris Descartes, 24 rue du Faubourg Saint-Jacques, 75014 Paris, France; benedicte.oules@yahoo.fr; 4 Université Côte d’Azur, INSERM, U1065, C3M, 06200 Nice, France; 5 Unité INSERM U1151 (Eq. 13), Faculté de Médecine Paris Descartes, 75993 Paris CEDEX 14, France; patriziapaterlini@gmail.com

**Keywords:** Alzheimer disease, amyloid β, amyloid precursor protein, BACE1, C83, C99, endoplasmic reticulum stress, neuroinflammation, truncated isoform of the sarco-endoplasmic reticulum Ca^2+^ ATPase 1 (S1T)

## Abstract

Dysregulation of the Endoplasmic Reticulum (ER) Ca^2+^ homeostasis and subsequent ER stress activation occur in Alzheimer Disease (AD). We studied the contribution of the human truncated isoform of the sarco-endoplasmic reticulum Ca^2+^ ATPase 1 (S1T) to AD. We examined S1T expression in human AD-affected brains and its functional consequences in cellular and transgenic mice AD models. S1T expression is increased in sporadic AD brains and correlates with amyloid β (Aβ) and ER stress chaperone protein levels. Increased S1T expression was also observed in human neuroblastoma cells expressing Swedish-mutated β-amyloid precursor protein (βAPP) or treated with Aβ oligomers. Lentiviral overexpression of S1T enhances in return the production of APP C-terminal fragments and Aβ through specific increases of β-secretase expression and activity, and triggers neuroinflammation. We describe a molecular interplay between S1T-dependent ER Ca^2+^ leak, ER stress and βAPP-derived fragments that could contribute to AD setting and/or progression.

## 1. Introduction

Alzheimer disease (AD) is a common age-dependent neurodegenerative disease that accounts for most of reported senile dementia cases [1]. The pathological features of AD include the formation of intracellular neurofibrillary tangles (NFTs) composed of hyperphosphorylated Tau, and the extracellular deposition of amyloid-β (Aβ) aggregates derived from the sequential processing of the β amyloid precursor protein (βAPP) by β- and γ-secretases [2]. At the cellular level, perturbations of the endoplasmic reticulum (ER) calcium (Ca^2+^) signalling have emerged as key features in AD-affected brains and models [3,4,5]. AD is also one of the prototypical proteinopathies characterized by cortical and hippocampal protein accumulation and aggregation, synaptic impairments, neuronal loss and consistent neuroinflammation [5,6,7].

Accumulation of unfolded proteins into the ER, as well as alteration of ER Ca^2+^ homeostasis, induce ER stress eliciting unfolded protein response (UPR) [8,9]. Chronic ER stress implicates the mitochondrial apoptotic pathway. Ca^2+^ fluxes from ER to mitochondria, tuned by physical interaction between the two organelles, emerged as a key regulator of this pathway [10,11,12]. In this context, we reported that the human truncated isoform of the Sarco-endoplasmic reticulum Ca^2+^ ATPase 1 (S1T) [13] triggers and amplifies ER stress response, leading to subsequent cell commitment to apoptosis through the control of Ca^2+^ mobilization from ER to mitochondria [14]. 

We report herein the increase of the human S1T protein expression in a cohort of human sporadic AD-derived post-mortem brains as well as in a cellular AD model. S1T elevation is mimicked by oligomeric Aβ treatment and in return enhances βAPP processing and the production of βAPP-derived toxic fragments (i.e., C99 and Aβ) in in vitro and in vivo AD models. Mechanistically, we find that S1T-mediated elevation of APP processing occurs through the up-regulation of BACE1 expression and enhanced activity, but not γ-secretase complex expression and activity. Interestingly, we also document that S1T expression induces a neuroinflammatory response in vitro and in vivo.

## 2. Material and Methods

### 2.1. Human Brain Samples

All procedures performed in studies involving human participants were in accordance with the ethical standards of the institutional and/or national research committee and with the 1964 Helsinki declaration and its later amendments or comparable ethical studies. Informed consent for tissue donation for research is obtained by the Brain Bank NeuroCEB and has been declared at the Ministry of Higher Education and Research (agreement AC-2013-1887) under their approval procedures.

Brain samples were obtained from the Brain Bank “NeuroCEB” run by a consortium of Patients Associations: ARSEP (association for research on multiple sclerosis), CSC (cerebellar ataxias), and France Parkinson. The consents were signed by the patients themselves or their next of kin in their name, in accordance with the French Bioethical Laws. Cases were anonymized, but information was provided regarding sex, age at death, and neuropathology (Table 1).

### 2.2. Lentiviruses Production

Lentiviruses were obtained by co-transfecting the transfer vector with two helper plasmids, ∆8.9 (packaging vector) and VSV-G (envelope vector) into Lenti-X 293 T cell line (632180; Clontech, Mountain View, CA, USA). Viral particles were purified from the culture medium, and viral titers were determined by p24 ELISA (VPK-107; Cell Biolabs, San Diego, CA, USA).

### 2.3. Cell Lines, Lentiviruses Infection, Transfections and Treatments

Human SH-SY5Y neuroblastoma cells (CRL-2266, ATCC), and murine microglial cells (BV2) [15] were cultured following the manufacturer’s instructions. SH-SY5Y cells stably expressing empty pcDNA3.1 vector (Control), or the human βAPP harboring the double Swedish mutations cDNA (APPswe: APPKM670/671NL) were already described [16]. 

SH-SY5Y and BV2 cells were transduced with Green, S1T-Green or SERCA1-Green lentiviruses (cloned in the lentiviral vector with an IRES- ZsGreen fluorescent tag (pHAGE-CMV-MCS-IRES-ZsGreen), under the control of CMV promoter) using five MOI (multiplicity of infection) (48h in BV2 cells and 72-96h in SH-SY5Y cells). 

We also used pcDNA 3.1, GFP targeted to the ER (ER-GFP), and GFP fused constructs SERCA1-GFP and S1T-GFP previously described [14]. Transient transfection of cells was carried out using Lipofectamine 2000 reagent (Invitrogen). Cells were treated overnight for the indicated time with thapsigargin (0.1, 0.5 or 1 µM), tunicamycin (10 µg/mL) or amyloid β oligomers (Aβo) 5 µM (as already described [17]). Human synthetic Aβ_1–42_ (Bachem AG, Switzerland) was prepared as already described [18]. 

### 2.4. Animal Models

All applicable international, national, and/or institutional guidelines for the care and use of animals were followed. All procedures performed in studies involving animals were in accordance with the ethical standards of the institution or practice at which the studies were conducted.

In vivo experiments were performed in accordance with the regulations of the Institutional Animal Care and Use Committee of the guidelines established by the European community council (Directive of 24th November 1986), and approved by Nice university Animal care and use Committee, and the National Council on animal care of the Ministry of Health (Project n°: NCE/2013-152).

Also, 3xTg-AD mice [19] were housed in the specific-pathogen-free animal facility with a 12:12h light/dark cycle and were given free access to food and water. 

For biochemical analyses, mice were anesthetized with a ketamine/xylazine (87 mg/mL and 13 mg/mL respectively, 1 mL/kg) mixture and transcardially perfused with PBS for 5 min. Brains were then isolated and stored in the RNA stabilization reagent (RNAlater, Qiagen) for 24h, and then dried and stored at −80 °C until use. For immunohistochemistry analyses, anesthetized mice were perfused with PBS and then with 10 mL PFA 4% solution. Brains were post-fixed in PFA 4% for 24 h. Fixed brains were cut on a vibratome (50 μm), or embedded in paraffin, and coronal sections were cut with a sliding microtome (8 μm).

### 2.5. Stereotaxic Injections and Treatments

Four-month-old non-transgenic (wild type: WT) and 3xTg-AD mice were anesthetized and then placed in a mouse head holder. Green or S1T-Green lentiviral particles were stereotactically injected bilaterally into the subiculum region (1 × 10^10^ viral particles, 2 μL per hemisphere) at the following coordinates (a/p, −1.7, m/l ±2.5, d/v, −3.8). After surgery, anesthesia was reversed with atipamezole (1 mg/kg). Mice were left under a heat lamp for allowing full recovery. Then they were injected with the analgesic buprenorphine at a dose of 0.05 mg/kg body weight and returned to a clean cage. Biochemical and immunohistochemistry analyses were carried out 4–5 months after lentiviral injections.

### 2.6. Protein Preparations and SDS-PAGE Analysis

Total proteins and microsomal and mitochondrial fractions were prepared as already described [14]. Intracellular Aβ peptide was detected as already described [16]. Proteins were resolved by 16.5% Tris/Tricine or 10–12% Tris/Glycine SDS-PAGE and transferred onto nitrocellulose membranes. Antibodies used are described in Supplementary Table S3. Proteins were visualized using enhanced chemiluminescence and acquired with LAS-4000 imaging system (Fujifilm).

### 2.7. Immunohistochemistry and Immunofluorescence

Human-derived hippocampal and temporal lobe sections and mice-derived slices embedded in paraffin were immunostained after a dewaxing procedure comprising sequential washing steps with xylene, ethanol and PBS. Sections were immunostained with S1T [13] or Iba1 antibodies after antigen unmasking using a Vector Antigen unmasking solution (Vector Laboratories). For 6E10 staining, sections were treated with formic acid (90% for 5 min). Serial slices were immunostained with S1T and 6E10 antibodies. Non-specific binding was blocked for 1 h in 5% BSA, 0.5% TBS-Triton X-100 solution. Sections were incubated at 4 °C overnight with primary antibodies (Supplementary Table S3). Sections were incubated with HRP-conjugated secondary antibodies (Jackson ImmunoResearch) followed by diaminobenzidine (DAB) substrate (Vector). Neuronal nuclei were detected with Cresyl violet.

### 2.8. In Vitro β-Secretase Assay

BACE-1 activity corresponding to the β-secretase inhibitor-sensitive fluorescence was monitored as already described [16,20].

### 2.9. Quantitative Real-Time PCR and Amplification S1T

Total RNA from cells and mice brains were extracted using the RNeasy mini kit and RNeasy universal, respectively (Qiagen, Germany) and then reverse-transcribed with GoScript™ Reverse Transcriptase (Promega, USA) using oligo-dT priming. Real-time PCR was performed in the Rotor-Gene6000 (Qiagen, Germany), using the SYBR Green detection protocol. Specific-gene primers are described in the supplementary material (Supplementary Table S4). Hot start touchdown amplification was performed to amplify S1T as already described [13] using primers on SERCA1 exon10 and exon13 sequences as described in Figure 2C and Supplementary Table S4.

### 2.10. Statistical Analyses

Data were expressed as means ± SEM. Sample size for each experiment is indicated in the figure captions. Data were analyzed with GraphPad Prism version 7.02 for Windows (GraphPad Software, La Jolla, CA, USA; www.graphpad.com). Data were first analyzed for normal distribution. We used the Mann-Whitney test when the two groups of variables have not passed the normality test. Groups of more than two variables that have passed normality test were analyzed by One-way ANOVA with Dunnett’s multiple comparison post-test. Correlations were analyzed with Spearman’s correlation coefficient. Significant differences are: * *p* < 0.05, ** *p* < 0.01, *** *p* < 0.001, **** *p* < 0.0001 and ns = non-significant.

## 3. Results

### 3.1. S1T Protein Expression is Increased in Human AD Brains

**Figure 1:**
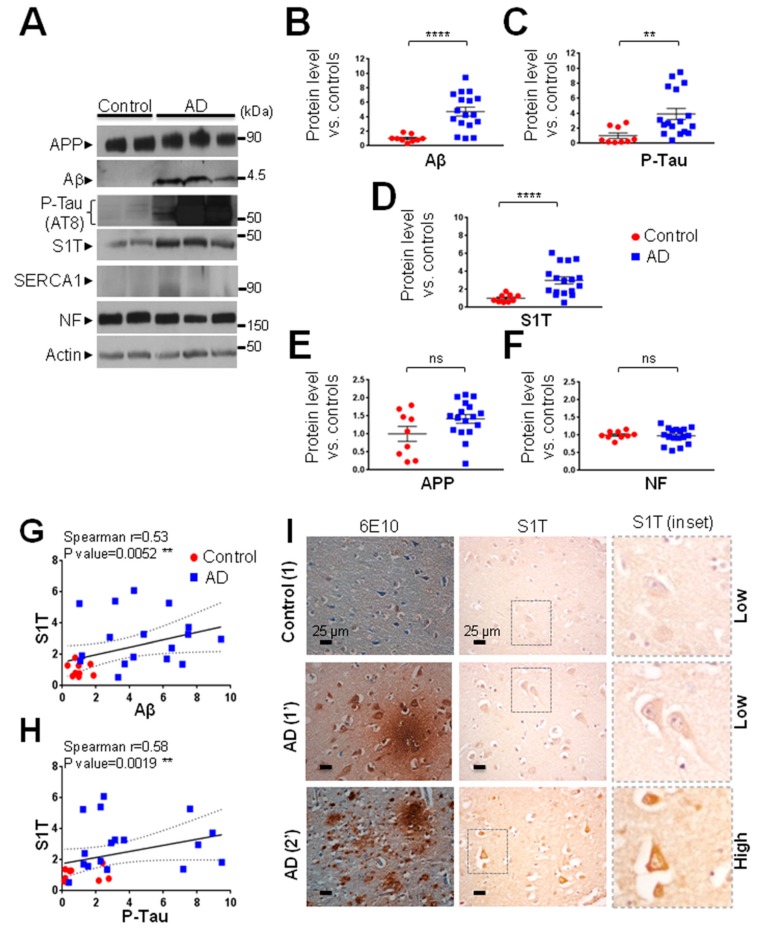
S1T expression is increased in human AD-affected brains. (**A**) Representative SDS-PAGE showing the expression pattern of S1T, APP, Aβ, Hyperphosphorylated Tau (P-Tau), Neurofilament (NF) in the temporal lobe of human AD brains (Braak’s stage IV, V and VI) (n = 17) as compared to aged-matched non-demented controls (n = 9). Demographic data and neuropathological status of brain samples are reported in Table 1. βAPP and Aβ were detected using 6E10 antibody (recognizing amino acids 1–16 of Aβ). Hyperphosphorylated Tau was detected by using AT8 antibody (recognizing phosphorylated (serine 202 and threonine 205) protein helical filament Tau, but not unphosphorylated Tau). Neurofilament (NF) and Actin were used as loading controls. S1T was detected using a homemade antibody recognizing a specific epitope in S1T protein directed towards the COOH-terminal 10 amino acid generated by exon 11 splicing [13]. SERCA1 was detected using an antibody recognizing N-terminal epitope. (**B**–**F**) Graphs represent means ± SEM of protein expression levels analyzed versus mean control values considered as 1. **** *p*-value < 0.0001, ** *p*-value < 0.01, and (ns) non-significant versus control using the Mann-Whitney test. (**G**,**H**) Correlation analyses between S1T expression and Aβ or P-Tau. Statistical Spearman r values and *p*-values were obtained using both control and AD individuals’ data. The regression line (dotted lines representing the 95 % confidence interval) is based on merged data. (**I**) Immunohistochemical detections of βAPP-derived fragments and of Aβ plaques with 6E10 antibody or of S1T in the temporal lobe of human AD (n = 9) and of aged-matched non-demented control brains (n = 4). Demographic data and neuropathological status of brain samples are reported in Table 1. S1T staining intensity and Aβ plaque type are reported in Supplementary Table S1. Nuclei were revealed using Cresyl Violet dye in 6E10 stained slices. The insets show S1T positive neurons. Scale bar 25 µm. Control (1), and AD (1′ and 2′) are referenced in Supplementary Table S1 and are representative samples harboring low or high S1T immunostaining.

We first analyzed the expression of S1T in a cohort of sporadic human AD brains and aged-matched non-demented controls (see Table 1 for demographic data and neuropathological status). As expected, we revealed significantly increased levels of Aβ peptide (Figure 1A,B) and of hyperphosphorylated Tau protein (P-Tau, Figure 1A,C) in AD brains while full-length βAPP (Figure 1A,E) remained unchanged. Neurofilament (NF) was also unchanged (Figure 1A,F). Importantly, by using an antibody specifically recognizing S1T protein but not full-length SERCA1 protein [13], we observed a significant increase of the expression of S1T in AD-affected brains (Figure 1A,D). We confirmed this increase with an anti-SERCA1 antibody recognizing the N-terminal sequence of both S1T and full-length SERCA1. We also revealed that full-length SERCA1 isoform appeared poorly expressed in both control and AD brains (Figure 1A). Additionally, we revealed that S1T expression levels correlated with both Aβ (Figure 1G) and P-Tau (Figure 1H) levels. These biochemical observations were strengthened by immunohistochemical analysis that revealed an increased staining of S1T in AD brain slices as compared to controls (Figure 1I, Table 1, and Supplementary Table S1). We classified S1T staining intensity as high or low (as shown in representative images in Figure 1I). These analyses showed that high-intensity staining of S1T was associated with focal Aβ deposits while low S1T staining was observed in samples displaying diffuse plaques (Supplementary Table S1), thus corroborating our biochemical observations linking high S1T expression to elevated levels of Aβ. We also observed an increased expression of S1T in the hippocampus of AD-derived brains as compared to controls (Supplementary Figure S1, and Supplementary Table S2). Overall, this set of data suggests a consistent increase of S1T isoform expression in various brain areas of a large cohort of late-stage sporadic AD-affected human brains.

### 3.2. ER Stress-Associated PERK-eIF2α-ATF4 Pathway is Modulated in Human AD Brains

**Figure 2:**
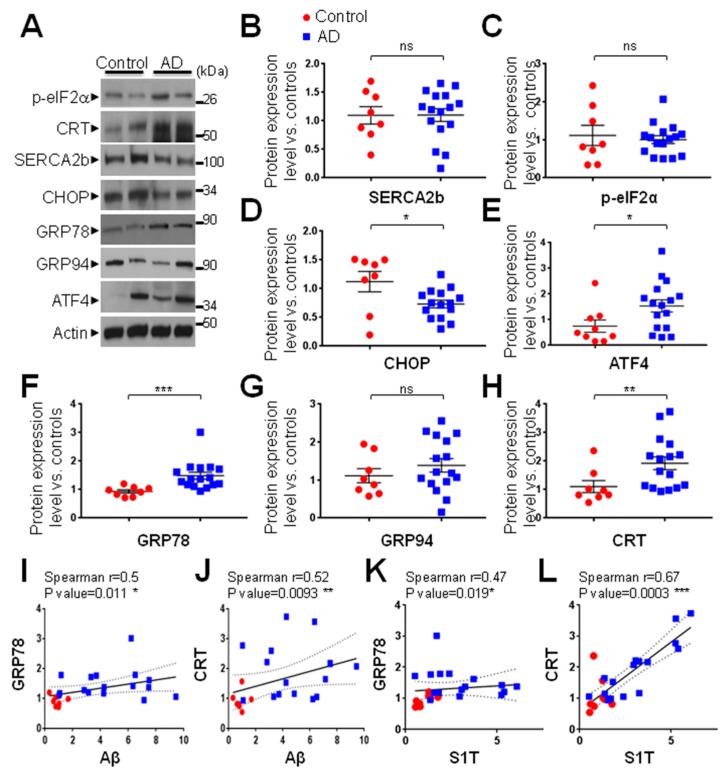
Expression of ER stress markers in human AD-affected brains. Demographic data and neuropathological findings of brain samples are reported in Table 1. (**A**) Representative SDS-PAGE showing the expression pattern of p-eIF2α, Calreticulin (CRT), SERCA2b, CHOP, glucose-regulated protein 78 (GRP78) and 94 (GRP94), and ATF4 in the temporal lobe of human AD-affected brains (n = 15–16) as compared to age-matched non-demented controls (n = 8–9). Actin was used as a loading control. (**B**–**H**) Graphs represent means ± SEM protein expression levels analyzed versus mean control values considered as 1. *** *p*-value < 0.001, ** *p*-value < 0.01, * *p*-value < 0.05, and (ns) non-significant versus control using the Mann-Whitney test. (**I**–**L**) Correlation analyses of GRP78 (**I**,**K**), and CRT (**J**,**L**) expression levels with Aβ (**I**,**J**) or S1T (**K**,**L**). Statistical Spearman r values and *p*-values were obtained using both control and AD individual’s data. The regression line (*dotted lines* representing the 95% confidence interval) are based on merged data.

We previously reported that S1T is induced under pharmacological and physiopathological ER stress conditions through the PERK-eIF2α-ATF4 pathway [14]. We thus analyzed the expression of ER stress markers belonging to this pathway in our sporadic human AD-derived brains cohort. We observed a significant increase of the expressions of ATF4 (Figure 2A,E), GRP78 (Figure 2A,F), and calreticulin (CRT) (Figure 2A,H) in AD brains as compared to controls, while the ubiquitous SERCA2b isoform (Figure 2A,B), p-eIF2α (Figure 2A,C), and GRP94 (Figure 2A,G) expressions remained unchanged. A slight but a significant decrease of CHOP protein was also observed in AD-derived brain samples (Figure 2A,D). We further revealed that increases of CRT and to a lesser extent GRP78, positively correlate with Aβ or S1T levels (Figure 2I–L) while correlation analyses between CHOP, ATF4, GRP94, and p-eIF2α proteins and Aβ or S1T levels failed to reach statistical significance (data not shown). All over, these data revealed for the first time an increased level of S1T protein in sporadic AD brains correlating with ER chaperones GRP78 and CRT (Figure 1 and Figure 2).

### 3.3. S1T Protein Expression is Increased in Human SH-SY5Y Cells Expressing APPswe or Treated by Oligomeric Aβ

**Figure 3:**
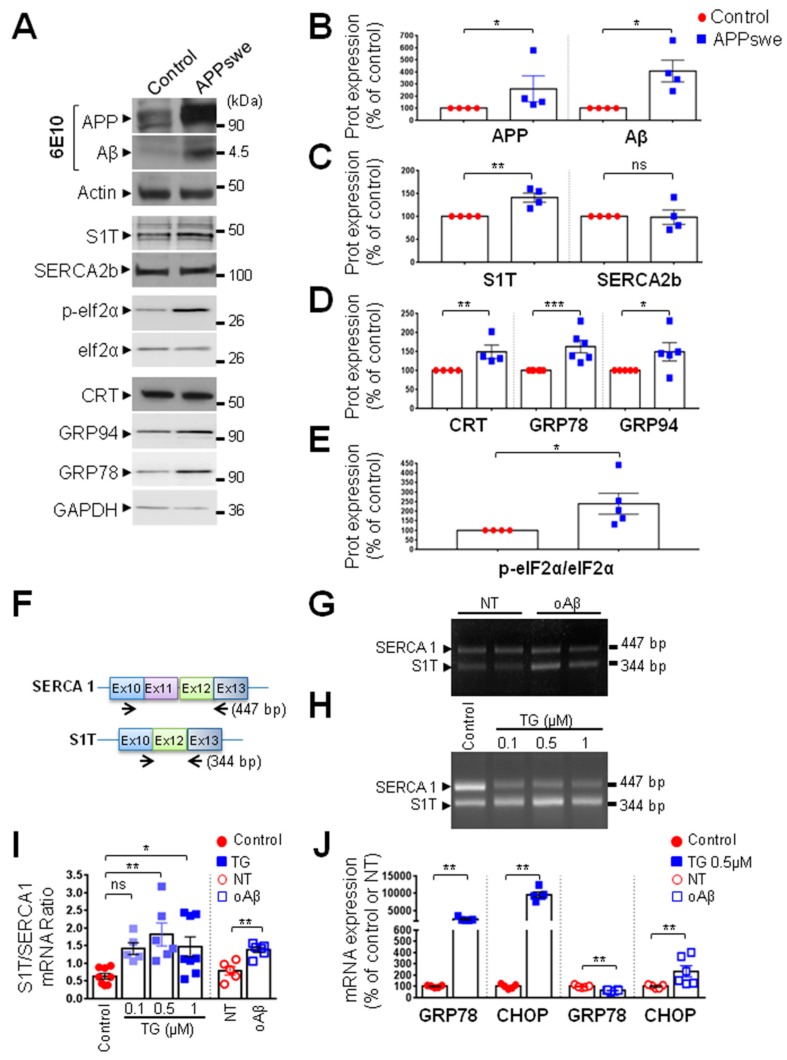
S1T expression is increased in the SH-SY5Y cells expressing APPswe and in SH-SY5Y cells treated with oligomeric Aβ peptides. (**A**) Representative SDS-PAGE showing the expression pattern of βAPP, Aβ as detected using 6E10 antibody, and of S1T and ER stress markers (SERCA2b, p-eIF2α, eIF2α, CRT, GRP78 and GRP94 in SH-SY5Y cells expressing pcDNA3.1 empty vector (Control) or APPswe construct (APPswe). Actin or GAPDH were used as loading controls. (**B**–**E**) Graphs represent means ± SEM of protein expression levels analyzed versus mean control values considered as 100% and obtained in 4–6 independent experiments. *** *p*-value < 0.001, ** *p*-value < 0.01, * *p*-value < 0.05, and (ns) non-significant versus control using the Mann-Whitney test. (**F**) Position of the primers (on exons 10 and 13) used to amplify SERCA1 transcripts with (447 bp: SERCA1) or without (344 bp: S1T) exon 11. (**G**,**H**) Representative gels of RT-PCR of SERCA1 and S1T expression patterns in SH-SY5Y cells non-treated (NT) or treated with oligomeric Aβ peptides (oAβ) (5 µM) (**G**), or treated with vehicle (Control) or thapsigargin (TG) (0.1, 0.5, and 1 µM) for 20 h (**H**). (**I**) Graph represents means ± SEM of S1T/SERCA mRNA ratio obtained in at least 4 independent experiments. ** *p*-value < 0.01, * *p*-value < 0.05, and (ns) non-significant versus control using the one-way ANOVA and Dunnett’s post-test, or versus NT using the Mann-Whitney test. (**J**) Quantitative RT-PCR of GRP78 and CHOP in SH-SY5Y cells treated with Aβ oligomers as in (**G**) or with 0.5 µM of TG (**H**). Genes were normalized for RNA concentrations with topoisomerase1 and GAPDH. The graph represents means ± SEM mRNA levels analyzed versus mean control or NT values taken as 100%. ** *p*-value < 0.01, versus control or NT using the Mann-Whitney test.

We then investigated expressions of S1T and ER stress markers in the neuroblastoma SH-SY5Y cell line stably expressing the Swedish mutated APP (APPswe: SH-SY5Y APPswe). This mutation was previously shown to enhance β-secretase-mediated cleavage of βAPP, thereby increasing productions of the βAPP C-terminal fragment C99, and subsequently Aβ peptides [16]. We confirmed the overexpression of full-length βAPP and enhanced production of Aβ in APPswe expressing cells as compared to mock-transfected control cells (Figure 3A,B). Importantly, we observed increased expressions of S1T protein (Figure 3A,C), CRT, GRP78, GRP94 (Figure 3A,D), and p-eIF2α (Figure 3A,E) in APPswe-expressing cells, while (as observed in human brains), the ubiquitous SERCA2b isoform expression remained unchanged (Figure 3A,C). Unlike what was observed in AD brains, we did not observe any significant modification of the expression of CHOP and ATF4 (data not shown).

Several lines of evidence indicate that oligomeric Aβ could act as a neurotoxic trigger in AD pathology through the induction of ER stress [21]. In accordance with previous studies, we used soluble synthetic Aβ42 oligomers (oAβ) as a toxic trigger [22,23], and specific set of primers allowing the amplification of both SERCA, and S1T transcripts (Figure 3F). S1T mRNA was increased in oAβ-treated SH-SY5Y cells as compared to non-treated (NT) cells (Figure 3G), as illustrated by an increased S1T/SERCA1 mRNA ratio (Figure 3I). We also observed an increase of CHOP transcript level and a slight but significant decrease of GRP78 mRNA upon oAβ treatment (Figure 3J). This agrees with previous studies showing that oAβ exposure modulates the expression of GRP78 and CHOP at the mRNA and protein levels in a time-dependent manner in SH-SY5Y cells [24]. As controls, we revealed that pharmacological ER stress induction by thapsigargin (TG) (a specific inhibitor of SERCA proteins) increased S1T/SERCA1 mRNA ratio (Figure 3H,I) as well as GRP78 and CHOP mRNAs levels (Figure 3J). Together, our data unraveled a consistent induction of S1T in neuroblastoma cells endogenously producing βAPP-derived fragments (APPswe cells) or exogenously treated with Aβ oligomers.

### 3.4. Expression of S1T Protein Enhances Amyloidogenic Processing of βAPP through Increased BACE1 Expression and Activity

**Figure 4:**
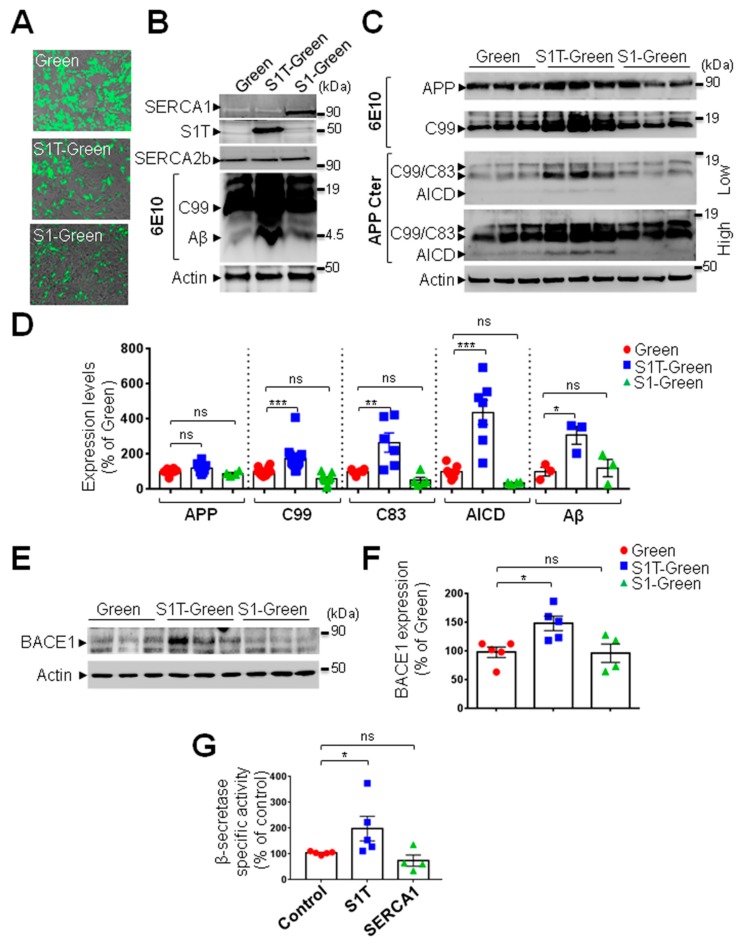
S1T overexpression enhances amyloidogenic βAPP processing. (**A**) Representative images of SH-SY5Y APPswe lentivirus-mediated stable Green, S1T-Green and SERCA1-Green cell lines. (**B**) Representative SDS-PAGE showing the expression patterns of SERCA1, S1T (detected with SERCA1 N-terminal antibody) and of β-secretase C-terminal APP-derived fragment (C99) and Aβ (detected with 6E10 antibody) in SH-SY5Y APPswe transduced cells. SERCA2b and actin were used as loading controls. (**C**) Low and high exposures of representative SDS-PAGE showing the expression pattern of full-length βAPP and C99 (detected with 6E10 antibody), and C99 and C83 (α-secretase-derived APP C-terminal fragment) and APP intracellular domain (AICD) as detected using an antibody recognizing APP C-terminal epitope (APP-Cter). Actin was used as a loading control. (**D**) The graph represents means ± SEM of protein expression levels analyzed versus mean control values (Green) considered as 100% and obtained in at least 4 independent experiments. *** *p*-value < 0.001, ** *p*-value < 0.01, * *p*-value < 0.5, and (ns) non-significant versus control using the one-way ANOVA and Dunnett’s post-test. (**E**) Representative SDS-PAGE showing BACE1 expression in SH-SY5Y APPswe cells transduced with Green, S1T-Green and SERCA1-Green lentiviruses. Actin was used as a loading control. (**F**) The graph represents means ± SEM of BACE1 expression level versus mean control values (Green) considered as 100% and obtained in at least 4 independent experiments. * *p*-value < 0.05, and (ns) non-significant versus Green using the one-way ANOVA and Dunnett’s post-test. (**G**) The graph represents the means ± SEM of the specific activity of β-secretase in SH-SY5Y APPswe cells transfected with ER-GFP (Control), S1T-GFP (S1T), SERCA1-GFP (SERCA1). β-secretase specific activity is shown as % of mean control values considered as 100%. * *p*-value < 0.05, and (ns) non-significant versus control using the one-way ANOVA and Dunnett’s post-test.

We generated lentivirus-based stable SH-SY5Y APPswe cell lines expressing S1T or SERCA1 cDNA simultaneously with the ZsGreen protein under IRES cassette. These constructs allow easy in situ detection after cell transduction (Figure 4A). S1T and SERCA1 overexpressions were also biochemically validated in microsomal protein extracts prepared from SH-SY5Y APPswe cells (Figure 4B).

We examined the potential modulation of βAPP processing by S1T by analyzing the level of intracellular βAPP-derived fragments. As shown in the representative SDS-PAGE (Figure 4B,C) and quantitative analyses (Figure 4D), S1T but not SERCA1 overexpression increased the production of all intracellular βAPP-derived fragments (C83, C99, Aβ, and AICD). Importantly, this was not due to increased full-length βAPP level (Figure 4C,D).

Further, we showed that S1T overexpression led to the accumulation of βAPP-derived fragments in the mitochondrial fraction (Supplementary Figure S2A,B).

We then investigated the potential implication of S1T in the modulation of non-amyloidogenic metabolism of βAPP. Neither S1T nor SERCA1 altered the production of the soluble sAPPα derived from the cleavage of βAPP by α-secretase (Supplementary Figure S3A,B). Accordingly, the expression of constitutive α-secretase (ADAM10) was not affected by S1T and SERCA1 (Supplementary Figure S3C,D).

Amyloidogenic metabolism of βAPP implies its sequential cleavage by β- and γ-secretases [25]. Thus the increase of C99 and Aβ peptide production upon S1T overexpression may be linked to the increased expression and/or activity of β- and/or γ-secretases. We showed that SH-SY5Y APPswe stably expressing S1T but not SERCA1 displayed increased expression of BACE1 (Figure 4E,F). Accordingly, we then revealed that S1T but not SERCA1 triggered a significant increase of the β-secretase specific activity in APPswe expressing cells (Figure 4G). The analyses of the expression of components of the γ-secretase complex [26] (Pen 2, Aph1, Nicastrin, PS1, and PS2) (Supplementary Figure S4A–F) revealed an unchanged level of PS1 (the catalytic core of the γ-secretase complex) in S1T expressing cells (Supplementary Figure S4A–F). Accordingly, we did not observe any significant modulation of the γ-secretase activity as analyzed in reconstituted membranes prepared from APPswe cells expressing S1T or SERCA1 proteins (Supplementary Figure S4G). Overall, these data reveal that enhanced β-amyloidogenic processing of βAPP upon S1T expression is specifically linked to the modulation of BACE1 expression and activity. Thus, the enhanced α-secretase-derived C83 fragment is likely due to an enhanced level of its precursor C99 fragment [27].

### 3.5. Lentiviral Expression of S1T Enhances β-Secretase-Mediated APP Processing In Vivo

**Figure 5:**
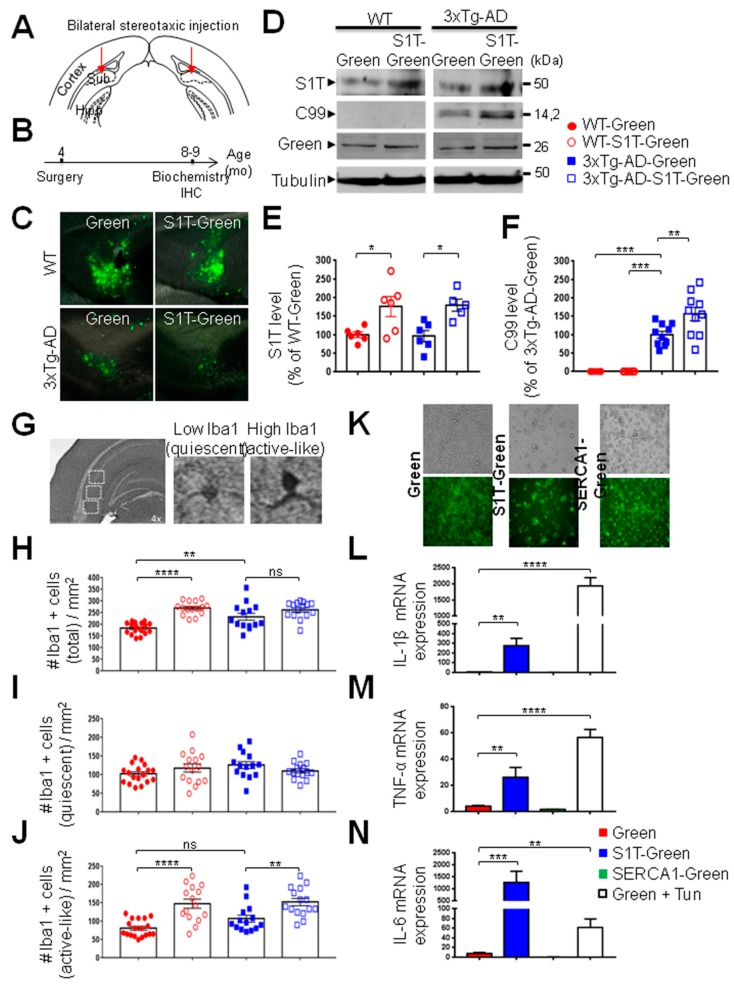
Lentivirus-mediated S1T delivery increases C99 production and triggers neuroinflammation in the 3xTg-AD mice. (**A**) Bilateral stereotaxic injection of lentiviruses (Green or S1T-Green) into the hippocampus (subiculum region) of 3xTg-AD and non-transgenic control mice (wild type: WT). (**B**) Timeline of lentiviral injection (mice aged 4 months-old) and of subsequent biochemical analysis and immunohistochemistry of mice (mice aged 8–9 months-old). (**C**) Representative coronal brain slices showing Green-positive cells (green) in injected mice. (**D**) Representative SDS-PAGE showing S1T, C99, and Green protein expression in injected mice. Tubulin was used as a loading control. (**E**) The graph represents the means ± SEM of S1T expression expressed as the percent of control WT mice considered as 100% (WT-Green (n = 6); WT-S1T-Green (n = 6), 3xTg-AD-Green (n = 6), and 3xTg-AD-S1T-Green (n = 5). * *p*-value < 0.05 versus WT-Green or 3xTg-AD-Green using the one-way ANOVA and Tukey’s post-test. (**F**) The graph represents the means ± SEM of C99 level versus the mean value in 3xTg-AD mice injected with Green lentivirus considered as 100%. *** *p*-value < 0.001, and ** *p*-value < 0.01 versus 3xTg-AD-Green using the one-way ANOVA and Dunnett’s post-test. (**G**) The number of total, quiescent, and activated microglia was recorded in three fields in the subiculum of injected mice. Quiescent microglia (low Iba1 staining and ramified microglia) and activated-like microglia (high Iba1 staining with thickened processes) were identified in separate fields in 3 different mice in each group. (**H**–**J**) Quantification of the total number (**H**), quiescent (**I**), and active-like Iba1 positive cells/mm^2^ (**J**). **** *p*-value < 0.0001, and ** *p*-value < 0.01, and (ns) non-significant versus WT-Green or 3xTg-AD-Green using the one-way ANOVA and Dunnett’s post-test. (**K**–**N**) Expression of S1T enhances the expression of proinflammatory cytokines in BV2 microglial cells. (**K**) Representative images showing BV2 cells infected with Green, S1T-Green or SERCA1-Green lentiviruses. (**L**–**N**) RT-PCR analysis of mRNA expression of IL1-β (**G**), TNF-α (**M**), and IL-6 (**N**). BV2 cells treated with tunicamycin (Tun) at 10 µg/mL, 16 h were used as control. The relative expression levels of mRNAs are represented as the means ± SEM versus the mean value in Green expressing cells (control) considered as 1 and obtained in 4 independent experiments in duplicates. **** *p*-value < 0.0001, *** *p*-value < 0.001, and ** *p*-value < 0.01 versus Green using the one-way ANOVA and Dunnett’s post-test.

The level of basal expression of S1T was not significantly enhanced in 3xTg-AD mice at different ages as compared to age-matched wild type mice (Supplementary Figure S5A,B). This observation was further confirmed in a second AD mice model, the Tg2576 mice (Supplementary Figure S5C). These data reveal a specific induction of S1T in human-derived cells and brains. Thus, we sought to investigate the impact of S1T overexpression in vivo through bilateral stereotaxic injection of lentiviral particles expressing S1T-Green or Green proteins in the subiculum of 3xTg-AD mice and their controls (Figure 5A,B). We confirmed the expected expression of Green protein (Figure 5C,D) and that of S1T in the subiculum of injected mice (Figure 5D,E). Interestingly, we observed that S1T overexpression enhanced the production of the C99 fragment in 3xTg-AD mice (Figure 5D,F).

### 3.6. Lentiviral Expression of S1T Protein Activates Microglia In Vivo and Enhances Proinflammatory Gene Expression in Cells

In addition to coordinating the expression of stress response genes during ER stress, the UPR initiates inflammatory pathways essential for the innate immune response [5,28]. Thus, we investigated the potential implication of S1T expression in neuroinflammation and focused on microglia activation. We analyzed proliferative activity and morphological changes (the main characteristic properties of active microglia) in both control and 3xTg-AD injected mice (as described in Figure 5A). In three areas of the subiculum (Figure 5G), we quantified the number of total microglia, quiescent ramified microglia characterized by low Iba1 intensity staining, and active-like microglia characterized by high Iba1 intensity staining with thickened processes and irregular cell bodies (see representative images in Figure 5G).

We observed an increased proliferative activity of microglia in 3xTg-AD mice as compared to control mice injected with Green lentivirus, as well as a significant increase of the number of total microglia in wild type mice upon S1T expression (Figure 5H). We also unraveled an increased active-like microglia in both wild type and 3xTg-AD mice injected with S1T lentivirus (Figure 5J), while the number of quiescent microglia between the different groups remained unchanged (Figure 5I). As a control, we performed the same analyses in a pharmacological model of ER stress obtained by intraperitoneal injection of tunicamycin in young wild type mice (Supplementary Figure S6). Tunicamycin treatment increases p-eIF2α, CHOP and calreticulin levels (Supplementary Figure S6A,B). Interestingly, as was seen upon S1T expression, we observed an enhanced number of total and activated microglia in tunicamycin-treated mice (Supplementary Figure S6C–E), while the number of quiescent microglia remained unchanged (Supplementary Figure S6F).

We further demonstrate the potential implication of S1T and of ER stress in neuroinflammatory response and revealed a significant increased expression of interleukin 1β (IL1-β) (Figure 5L), tumor necrosis α (TNF-α) (Figure 5M), and interleukin 6 (IL-6) (Figure 5N) in BV2 cells upon S1T-Green expression (Figure 5K), or treatment with tunicamycin, but not upon SERCA1-Green expression (Figure 5K-N). Importantly, we also reported enhanced IL1-β, TNF-α, and IL-6 mRNA levels in vivo in tunicamycin-treated mice (Supplementary Figure S5G). Altogether, both in vivo and in vitro approaches demonstrate a regulation of neuroinflammatory response under ER stress conditions triggered by S1T expression or pharmacological challenge.

## 4. Discussion

Several studies have reported that UPR occurs relatively early in AD and that ER stress sensors PERK and IRE1α are activated in human AD brains [29,30]. We previously demonstrated that the human S1T isoform is induced under pharmacological and physiopathological ER stress conditions leading to ER stress amplification [14]. UPR activation has been proposed to be linked to intracellular Aβ accumulation [31]. Accordingly, we reveal herein that S1T is upregulated in a cellular AD model overproducing βAPP-derived catabolites. Importantly, biochemical data indicate that enhanced human S1T expression correlates with Aβ load in human AD-affected brains and that S1T high immunostaining is selectively observed in human AD cases harboring focal Aβ plaques. We further demonstrated that S1T expression is induced by exogenous application of Aβ oligomers in SH-SY5Y cells.

S1T is induced through the activation of the PERK-eIF2α-ATF4-CHOP pathway [14]. We observed an increased expression of p-eIF2α in SH-SY5Y APPswe cells thus confirming previous studies reporting increased phosphorylation of eIF2α in both in cellular and in vivo models expressing the APPswe mutation [32]. However, we observed discrepancies for p-eIF2α, ATF4 and CHOP expressions between SH-SY5Y APPswe cells and AD brains. These data may indicate distinct time-dependent regulations of PERK-eIF2α-ATF4-CHOP pathway in cells and in vivo AD study models. Accordingly, we and others reported that UPR genes show a dose- and time-dependent induction, transient decline, and re-induction kinetics under pharmacological ER stress stimuli [14,33]. Moreover, elevations of p-PERK, p-IRE1, and p-eIF2α in human AD were demonstrated to be brain-area dependent [34], associated with abnormally phosphorylated Tau [30,34,35], and likely more involved at the early stages of AD pathology [7,30]. Thus, cellular and mice AD models could not fully recapitulate the pathological alterations observed in human AD progression. According to this possibility, the induction of ER stress response in AD mice models has been recently questioned [36,37,38], and we did not reveal any change in the expression of endogenous S1T, nor of the GRP78 level in the 3xTg-AD mice at different ages (Supplementary Figure S5A,B).

We reveal that S1T overexpression increases the accumulation of intracellular βAPP-derived fragments (C-terminal fragments: CTFs (C99, and C83), Aβ, and AICD) in the mitochondrial fraction of APPswe cells. We previously documented an Aβ and βAPP-CTFs accumulation in mitochondria-associated membranes (MAMs) in both in vitro and in vivo AD models [22]. Other studies showed that these fragments harbor toxic properties toward mitochondria [21,39]. Thus, we propose that S1T-mediated intracellular/mitochondrial accumulations of βAPP-derived toxic fragments may promote mitochondrial-dependent cell death.

S1T-associated pro-amyloidogenic phenotype is mechanistically linked to increased BACE1 expression and activity with no alterations of α- and γ-secretases. Several lines of evidence indicated that enhanced phosphorylations of PERK and eIF2α in the AD brain are associated with increased amyloidogenic βAPP processing [32,40,41] through increased BACE1 expression [42,43]. Altogether, and as reviewed in [44], our data strengthen the molecular link between ER stress and BACE1.

**Figure 6:**
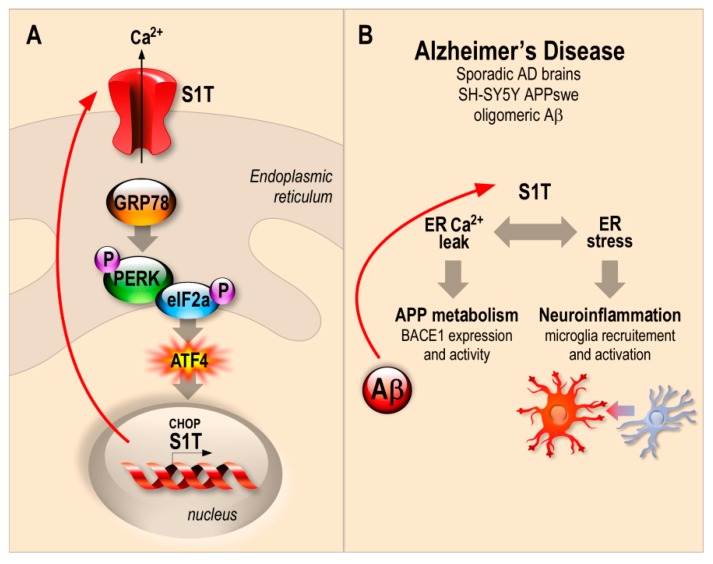
Schematic representation of the molecular interplay between S1T-dependent ER Ca^2+^ leak, ER stress, βAPP processing, and neuroinflammation likely contributing to AD setting and/or progression. (**A**) The human truncated isoform of the sarco-endoplasmic reticulum Ca^2+^ ATPase 1 (S1T) expression is induced under ER stress conditions through the PERK-eIF2α-ATF4-CHOP pathway, and triggers and amplifies ER stress response through the control of Ca^2+^ mobilization from ER. (**B**) S1T is upregulated in sporadic AD brains and in cellular models overproducing βAPP fragments (SH-SY5Y APPswe). S1T expression is also induced by exogenous oligomeric Aβ and enhances in turn βAPP processing through enhanced BACE1 expression and activity and triggers microglia recruitment and activation in vitro and in vivo.

The three UPR branches converge on activating NF-κB, thus driving the expression of cytokines (TNFα, IL-1, IL-6 and IL-8) [28], accordingly, we demonstrate herein that S1T overexpression, as well as tunicamycin treatment, induce the expression of proinflammatory cytokines and increase proliferation and active microglia. Tunicamycin induces ER stress response and enhances S1T expression thus exacerbating ER stress response and ER Ca^2+^ leak [14]. Tunicamycin also activates gangliosides biosynthesis [45], thereby interfering with APP processing [46]. A recent study also reported that in vivo tunicamycin administration induced spatial memory deficits and impairments of synaptic plasticity in rats [47]. These data are in agreement with our observations linking S1T in particular and ER stress response in general to AD development and/or progression. Overall, our study provides evidence that S1T expression may impact disease development through the upregulation of β-secretase-mediated βAPP processing and neuroinflammatory response using both cellular and in vivo study models. Thus, we propose an amplifying mechanism in a positive feedback loop between S1T and amyloidogenic βAPP processing likely accounting for the enhanced ER stress and neuroinflammation occurring in AD (Figure 6).

## Figures and Tables

**Table 1:** Demographic data and neuropathological findings related to human brain samples used in SDS-PAGE analyses (temporal lobe), and brain-derived slices used in immunohistochemistry analyses (T1 region of the temporal lobe).

	Age (years)	Gender	PMD (h)	Braak’s NFT Stage ^#^
Brain samples used in SDS-PAGE analyses				
Control	84	Male	32	-
Control	71	Female	15	-
Control	55	Male	25	-
Control	78	Male	35	-
Control	61	Male	20	-
Control	74	Female	49	-
ALS	62	Female	44	-
ALS	55	Male	21	-
ALS	62	Female	21	-
AD	81	Female	60	IV
AD	80	Male	23	V
AD	65	Male	70	V
AD	80	Female	51	V
AD	84	Female	81	V
AD	78	Female	18	VI
AD	65	Female	41	VI
AD	81	Male	19	VI
AD	75	Female	7	VI
AD	89	Female	26	VI
AD	93	Female	21	VI
AD	91	Female	34	VI
AD	55	Female	58	VI
AD	81	Female	NA	VI
AD	79	Male	31	VI
AD	82	Female	NA	VI
AD	86	Male	32	VI
Brain-derived slices used in immunohistochemistry analyses				
Control	62	Female	NA	-
ALS	62	Female	NA	-
ALS	55	Male	NA	-
ALS	68	Female	NA	-
AD	77	Female	NA	III
AD	55	Female	NA	IV
AD	65	Male	NA	V
AD	84	Female	NA	V
AD	82	Male	NA	V
AD	80	Male	NA	V
AD	86	Male	NA	VI
AD	63	Female	NA	VI
AD	79	Male	NA	VI

PMD: Post mortem delay, h: hours, NA: Not available. Controls are brain samples isolated from patients diagnosed as negative for several neuropathologies. ALS are control brains samples diagnosed as negative for AD pathology obtained from post-mortem patients diagnosed with amyotrophic lateral sclerosis (ALS). ^#^ Braak and Braak’s NFT (neurofibrillary tangle: Tau-related pathology) stage, (-) means no NFT detection.

## Supplementary Materials

The following are available online at https://www.mdpi.com/2073-4409/8/12/1539/s1.
