# Supplementary material for: Upregulation of the Sarco-Endoplasmic Reticulum Calcium ATPase 1 Truncated Isoform Plays a Pathogenic Role in Alzheimer’s Disease
Source: Cells. 2019 Nov 28;8(12):1539. doi: 10.3390/cells8121539 (PMC6953121; doi:10.3390/cells8121539)
Supplement: Supplementary file 1 [file cells-08-01539-s001.pdf]

Supplementary data

Supplementary material and methods

In vitro *γ-secretase assay*

In vitro γ-secretase assay was assessed as already described [44]. Twenty µg of each subcellular fraction were resuspended in solubilization buffer (150mM sodium citrate pH 6.4 containing 3-[(3-cholamydopropyl) dimethylammonio]-2-hydroxy-1-propanesulfonate 1% (v/v)) supplemented with protease inhibitor mixture. All steps were performed at 4°C. Solubilized membranes were diluted once with sodium citrate buffer (150 mM pH 6.4), and with reaction buffer (150mM sodium citrate pH 6.4, 20mM dithiothreitol, 0.2mg/ml BSA, 1mg/ml egg phosphatidyl choline and 50µg/mL recombinant C100-FLAG). The resulting reaction mixes were then either incubated over constant agitation for 16h at 37°C or stored at 4°C (negative controls). Samples were then supplemented with 2x Tris-Tricine loading buffer, boiled for 5 min and subjected to 16.5% Tris-Tricine SDS-PAGE.

Tunicamycin treated mice

We used (C57BL6) Wild-type mice purchased from Charles River. Mice were housed in the specific-pathogen-free animal facility with a 12:12h light/dark cycle and were given free access to food and water. The experimental procedure was in accordance with the European Communities Council Directive of 24 November 1986 (86/609/EEC) and local French legislation. Mice (12-14 week-old) were daily injected intraperitoneally for 3 days with either vehicle (0.03% DMSO, 0.02M Na_2HPO_4 buffer containing 150mM dextrose) or tunicamycin (1mg/kg, Merck) dissolved in the same vehicle. Brains were collected for biochemistry, RNA extraction, and immunochemistry.

Supplementary Figures, legends to Figures and Tables

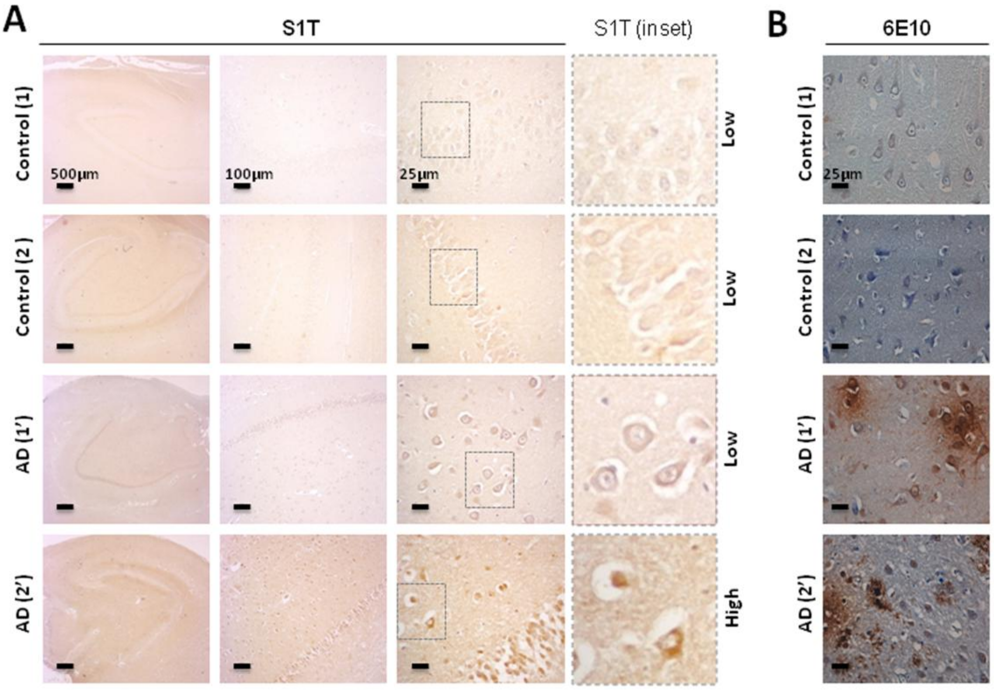

Supplementary Figure 1. Representative images showing S1T expression in the hippocampus of controls and human AD brain slices. S1T staining intensity and Aβ plaque type were analyzed in AD (n=6) and aged-matched non-demented control brains (n=4). Demographic data and neuropathological status of brain samples are reported in supplementary table 2. Nuclei were stained with Cresyl Violet dye on 6E10 stained slices. (A) S1T staining is shown in Control (1), Control (2), AD (1′) and AD (2′) (referenced in supplementary table 2) and are representative samples harboring low or high S1T immunostaining. Three different magnifications are shown. Scale bars 500, 100, and 25µm. The insets show S1T positive neurons. (B) Representative 6E10 staining in the brain sections obtained from the same patients.

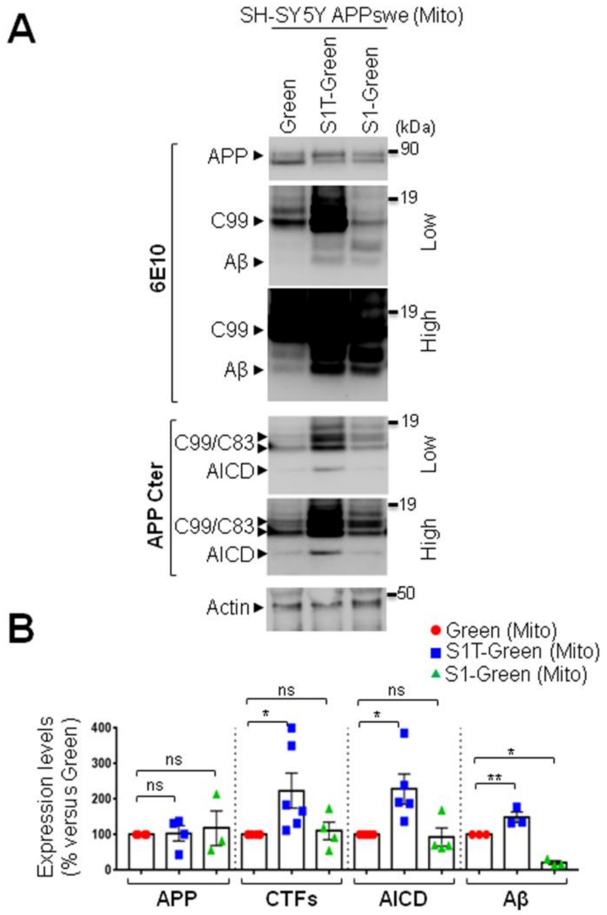

Supplementary Figure 2. S1T overexpression enhances βAPP-derived catabolites accumulation in the mitochondria. (A) βAPP and its-derived catabolites were detected in the mitochondrial enriched fraction isolated from SH-SY5Y APPswe cells. βAPP, the β-secretase-derived C-terminal APP fragment (C99), and Aβ were detected using 6E10 antibody. APP CTFs (C99 and α-secretase C-terminal APP derived fragment (C83)) and APP intracellular domain (AICD) were detected using an antibody recognizing the βAPP C-terminal epitope (APP-Cter). Low and high exposures of representative SDS-PAGE are shown. Actin was used as loading control. (B) The graph represents means±SEM of APP, CTFs (C99, and C83), AICD and Aβ expression levels analyzed versus mean control values (Green) considered as 100% and obtained in more than 3 independent experiments. ** *p-value* <0.01, * *p-value* <0.5, and (ns) non-significant versus control using the one-way ANOVA and Dunnett's post-test.

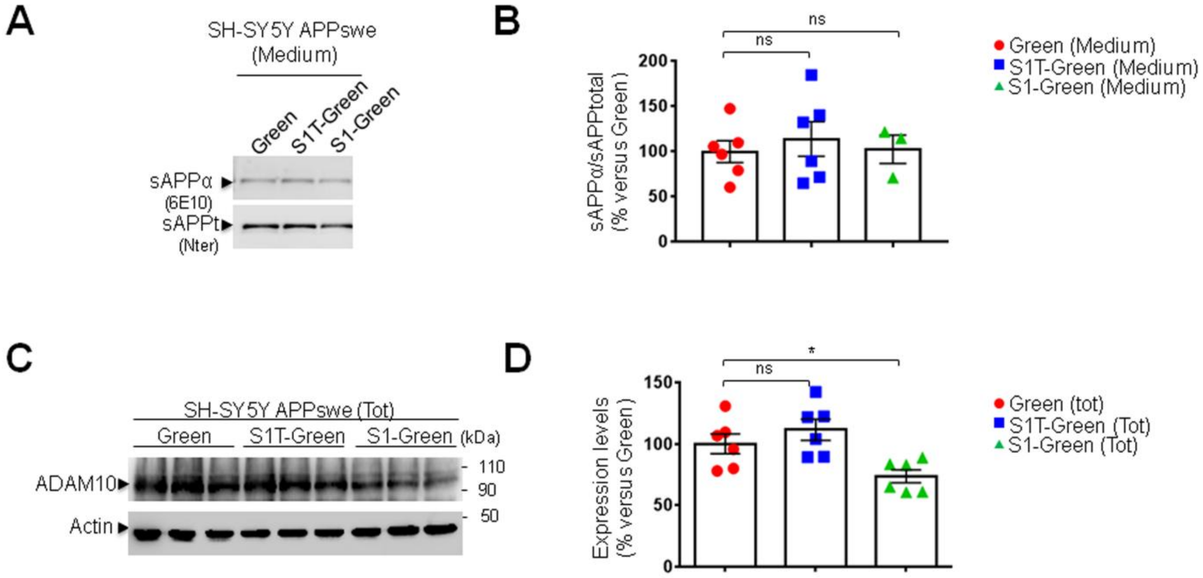

Supplementary Figure 3. S1T overexpression does not alter α-secretase expression and activity. (A) Representative SDS-PAGE showing the level of secreted APPα fragment (sAPPα) detected by 6E10 antibody versus total (sAPPα and sAPPβ) fragments (sAPPt) detected using an antibody recognizing APP N-terminal epitope (APP-Nter). The same concentration of secreted proteins (in the medium) was loaded in each condition. (B) The graph represents the mean±SEM of sAPPα/sAPPt ratio versus the mean control values (Green) considered as 100% and obtained in at least 3 independent experiments. (ns) non-significant versus Green using the one-way ANOVA and Dunnett's post-test. (C) Representative SDS-PAGE showing ADAM10 (constitutive α-secretase) expression in SH-SY5Y APPswe cells transduced with Green, S1T-Green and SERCA1-Green lentiviral vectors as in Figure 3A. Actin was used as loading control. (D) The graph represents the mean±SEM of ADAM10 expression level obtained versus the mean control values (Green) considered as 100% and obtained in 6 independent experiments. * *p-value* <0.05, and (ns) non-significant versus Green using the one-way ANOVA and Dunnett's post-test.

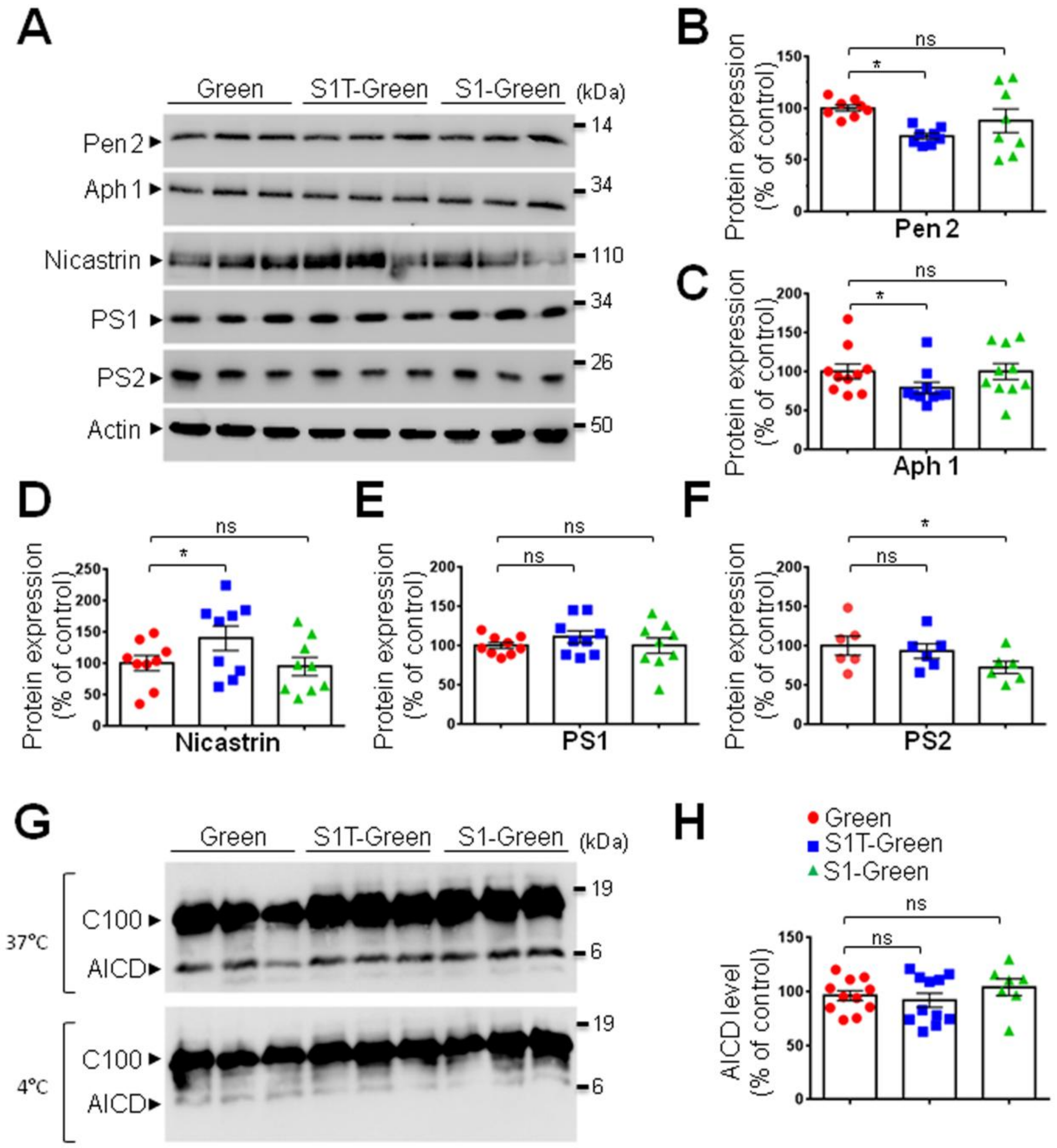

Supplementary Figure 4. S1T overexpression does not affect the expression and activity of γ-secretase complex. (A) Representative SDS-PAGE showing the expression of γ-secretase complex (Pen2, Aph1, Nicastrin, PS1 and PS2) in SH-SY5Y APPswe cells transduced with Green, S1T-Green or SERCA1-Green lentiviruses. (B-F) Graphs represent mean±SEM expression levels of Pen2 (F), Aph1 (G), Nicastrin (H), PS1 (I) and PS2 (J) versus mean control values (Green) considered as 100% and obtained in at least 4 independent experiments. * *p-value* <0.05, and (ns) non-significant versus Green using the one-way ANOVA and Dunnett's post-test. (G) Cell-free AICD production from recombinant C100 peptide performed at 37°C or 4°C in the presence of membrane extracts isolated from APPswe cells expressing Green, S1T-Green or SERCA1-Green. C100 and AICD were detected using APP-Cter antibody. (H) The graph represents mean±SEM AICD level versus mean control values (Green) considered as 100% and obtained in at least 7 independent experiments. (ns) non-significant versus Green using the one-way ANOVA and Dunnett's post-test.

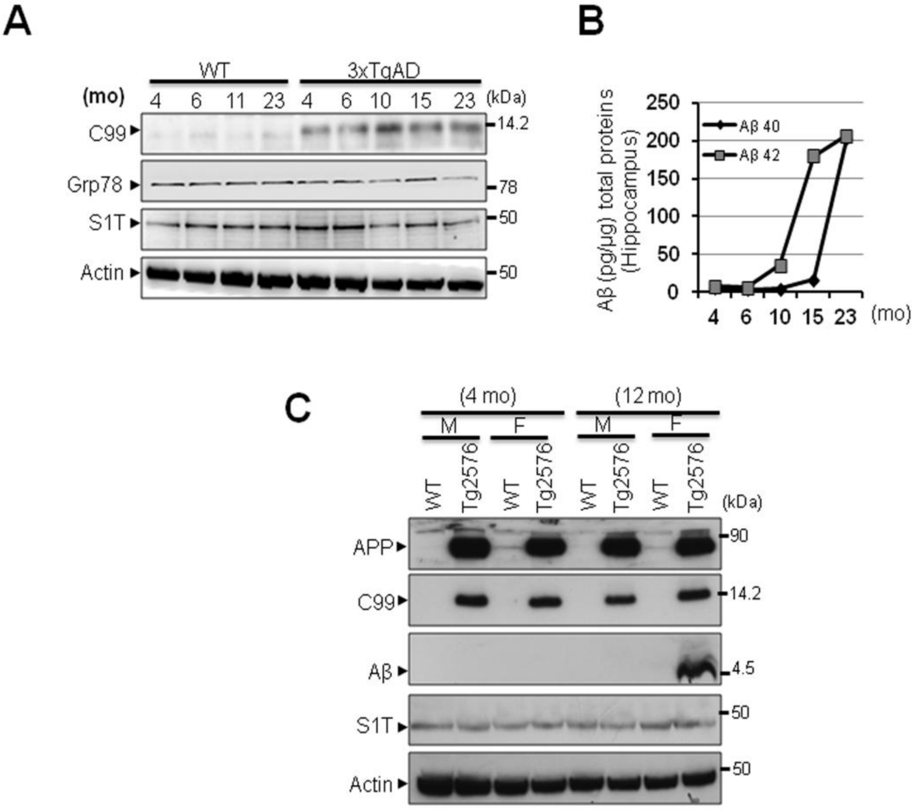

Supplementary Figure 5. S1T expression is unchanged in mice AD brains. (A) SDS-PAGE showing the expression pattern of C99, GRP78, and S1T, in the hippocampus of WT (aged 4, 6, 11, and 23 months) and 3xTg-AD (aged 4, 6, 10, 15,, and 23 months) mice. (B) Total (soluble and insoluble) Aβ40 and Aβ42 were quantified in 3xTg-AD by ELISA. (C) SDS-PAGE showing the expression pattern of full length APP, C99, Aβ, and S1T, in the cortex of male and females of WT and Tg2576 (aged 4, and 12 months) mice.

APP, C99 and Aβ were detected using 6E10 antibody. S1T was detected using a homemade antibody recognizing a specific epitope in S1T protein directed towards the COOH-terminal 10 amino acid generated by exon 11 splicing [13]. Actin was used as loading control (A, C).

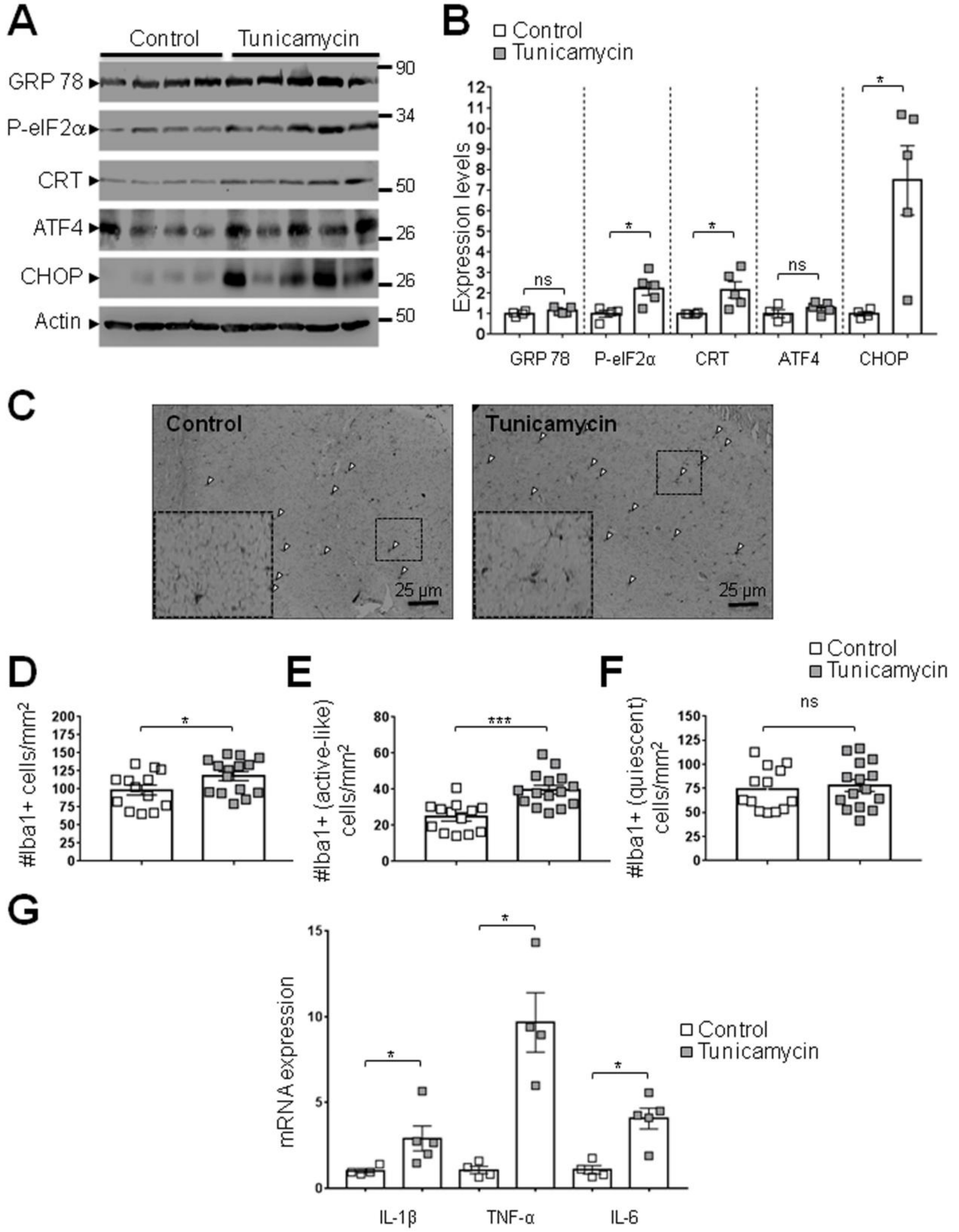

Supplementary Figure 6. Tunicamycin administration induces ER stress and neuroinflammation *in vivo*. (A) Representative SDS-PAGE showing the expression pattern of glucose-regulated protein 78(GRP78), p-eIF2α, Calreticulin (CRT), ATF4, and CHOP in the brain of control (n=4) and tunicamycin-treated mice (n=5). Actin was used as loading control. (B) Graph represents mean±SEM protein expression levels analyzed versus mean control values considered as 1. * *p-value* <0.05, and (ns) non-significant versus control using the Mann-Whitney test. C/ Representative images showing immunohistochemical staining of Iba1 in control or Tunicamycin-treated mice. Scale bar, 25 µm. (D-F) The number of total (D), active-like (E), and quiescent (F) microglia were recorded in three fields in the hippocampus of treated mice as described in Figure 5. *** *p-value* <0.001, and * *p-value* <0.05, and (ns) non-significant versus control mice using the one-way ANOVA and Dunnett's post-test. G/ RT-PCR

analysis of the mRNA expression of IL1-β, TNF-*α*, and IL-6 in the brain of control (n=4) and tunicamycin-treated mice (n=5). Genes were normalized for RNA concentrations with topoisomerase1 and GAPDH. The relative expression levels of mRNAs are represented as the mean±SEM versus the mean value in control mice. * *p-value* <0.05 versus control using the one-way ANOVA and Dunnett's post-test.

Supplementary Table 1: demographic data and neuropathological findings related to human temporal lobe (T1) samples used in immunohistochemistry analyses

	Age (years)	Gender	Braak's NFT stage[#]	Aβ deposits[##]	S1T staining[###]
Control	62	Female	-	0	Low
ALS	62	Female	-	0	Low
ALS (1)	55	Male	-	0	Low
ALS	68	Female	-	DP	Low
AD	77	Female	III	FP	High
AD	55	Female	IV	FP	High
AD (1′)	65	Male	V	DP	Low
AD	84	Female	V	FP	High
AD	82	Male	V	FP	High
AD	80	Male	V	FP	High
AD (2′)	86	Male	VI	FP	High
AD	63	Female	VI	FP	High
AD	79	Male	VI	FP	High

Control is brain sample isolated from a patient diagnosed as negative for several neuropathologies. ALS are control brains samples obtained from post-mortem patients diagnosed with Amyotrophic lateral sclerosis (ALS). [#] Braak and Braak's NFT (neurofibrillary tangle: tau-related pathology) stage, (-) no NFT detection. [##] Aβ deposits (0: no deposits, DF: diffuse plaque staining, FP: focal plaque staining). [###] S1T immunostaining intensity was semiquantitatively classified as low or high as shown in representative images in Fig.1G. Representative images of S1T staining and of Aβ plaques immunostaining in the temporal lobe (T1) of control (1), AD (1′), and AD (2′) are shown in Fig. 1G.

Supplementary Table 2: demographic data and neuropathological findings related to human hippocampus (H) samples used in immunohistochemistry analyses

	**Age (years)**	**Gender**	**Braak's NFT stage**[#]	**Aβ deposits**[##]	**S1T staining**[###]
Control (1)	62	Female	-	0	Low
ALS	62	Female	-	0	Low
ALS (2)	55	Male	-	0	Low
ALS	68	Female	-	DP	Low
AD	90	Male	III	FP	High
AD	84	Female	V	ND	Low
AD (1′)	65	Male	V	DP	Low
AD	63	Female	VI	FP	Low
AD (2′)	86	Male	VI	FP	High
AD	79	Male	VI	FP	High

Control is brain sample isolated from a patient diagnosed as negative for several neuropathologies. ALS are control brains samples obtained from post-mortem patients diagnosed with Amyotrophic lateral sclerosis (ALS). [#] Braak and Braak's NFT (neurofibrillary tangle: tau-related pathology) stage, (-) no NFT detection. [##] Aβ deposits (0: no deposits, DF: diffuse plaque staining, FP: focal plaque staining, ND: not determined). [###] S1T immunostaining intensity was semiquantitatively classified as low or high as shown in representative images in supplementary Fig.1. Representative images of S1T and Aβ plaques immunostaining in the hippocampus of control (1), and control (2), AD (1′), and AD (2′) are shown in supplementary Fig. 1.

Supplementary Table 3: List of antibodies used in the study

**Antibody**	**Host**	**Supplier**	**WB dilution**	**IF dilution**
6E10 (1-16 aa of Aβ)	Mouse	Covance	1/1000	1/2000
β-APP CTFs (A8717)	Rabbit	Sigma Aldrich	1/1000	
APP N-terminal (22C11)	Mouse	Millipore	1/1000	
ATF4 (CREB-2) sc-200	Rabbit	Santa Cruz Biotechnologies	1/1000	
GRP78 (ab21685)	Rabbit	abcam	1/2000	
GRP94 (ab18055)	Rabbit	abcam	1/2000	
Calreticulin (C17)	Goat	Santa Cruz Biotechnologies	1/2000	
eIF2α	Rabbit	Cell Signalling	1/2000	
P-eIF2α (119A11)	Rabbit	Cell Signalling	1/2000	
Iba1	Rabbit	Wako, Fujifilm	-	1/1000
Aph1 (PA5-20318)	Rabbit	Thermo Scientific	1/2000	
PEN2 N-terminal	Rabbit	Millipore	1/2000	
BACE-1 (D10E5)	Rabbit	Cell signaling	1/2000	
Nicastrin	Rabbit	Sigma Aldrich	1/2000	
zsGreen	Rabbit	Clontech	1/2000	
ADAM10	Rabbit	Euromedex	1/1000	
SERCA2b (IID8)	Mouse	Affinity Bioreagents	1/2000	
SERCA1	Rabbit	Santa Cruz Biotechnologies	1/1000	1/50
CHOP (9C8)	Mouse	Thermo scientific	1/1000	
Pan-Neurofilament (SMI-311)	Mouse	abcam	1/1000	
TAU(AT8)	Mouse	Pierce	1/1000	
β-Actin	Mouse	Sigma Aldrich	1/5000	
GAPDH (A01)	Mouse	abnova	1/2000	
S1T	Rabbit	Homemaid [13]	1/1000	1/50

Supplementary Table 4: primer sequences used in the study

Gene	Primer	Sequences
GAPDH human	Sens	5'-AGCCACATCGCTCAGACAC-3'
	Antisens	5'-GCCCAATACGACCAAATCC-3'
GAPDH mouse	Sens	5'-AAGAGGGATGCTGCCCTTAC-3'
	Antisens	5'-CCATTTTGTCTACGGGACGA-3'
Topoisomerase human	Sens	5'-CCCTGTACTTCATCGACAAGC-3'
	Antisens	5'-CCACAGTGTCCGCTGTTTC-3'
Topoisomerase mouse	Sens	5'-TGCCTCCATCACACTACAGC-3'
	Antisens	5'-CGCTGGTACATTCTCATCAGG-3'
CHOP human	Sens	5'-CGACAGAGCCAAAATCAGAGC-3'
	Antisens	5'-CAGTGTCCGAAGAGAAAGGCAA-3'
GRP78 human	Sens	5'-GAAGCCCGTCCAGAAAGTGT-3'
	Antisens	5'-TTATGCCACGGGATGGTTCC-3'
S1T human	Sens	5'-TATCATTGACAAGGTGGATGGGGACAT-3'
	Antisens	5'- CTTGACAAACATCTTGTTGCCCACAGCA-3'
Il1 beta mouse	Sens	5'- TGTAATGAAAGACGGCACACC-3'
	Antisens	5'-TCTTCTTTGGGTATTGCTTGG-3'
Il6 mouse	Sens	5'-CTACCAAACTGGATATAATCAGGA-3'
	Antisens	5'-CCAGGTAGCTATGGTACTCCAGAA-3'
TNF alpha mouse	Sens	5'-TCTTCTCATTCCTGCTTGTGG-3'
	Antisens	5'-GAGGCCATTTGGGAACTTCT-3'
